# Effectiveness of Ready-to-Use Therapeutic Food Among Children With Protein-Calorie Malnutrition

**DOI:** 10.7759/cureus.25872

**Published:** 2022-06-12

**Authors:** Geeta Bai, Arit Parkash, Vikash Kumar, Kirpal Das, Unica Akhtar, Arti .

**Affiliations:** 1 Pediatrics, National Institute of Child Health, Karachi, PAK; 2 Pediatric Gastroenterology & Hepatology, National Institute of Child Health, Karachi, PAK; 3 Internal Medicine, The Brooklyn Hospital Center, New York, USA; 4 Internal Medicine, Khair Pur Medical College and People's University of Health Sciences for Women, Khair Pur, PAK; 5 Nursing, National Institute of Child Health, Karachi, PAK; 6 Pediatrics, CMC Children Hospital, Larkana, PAK

**Keywords:** pakistan, weight gain, mid-upper arm circumference, ready to use therapeutic food, protein calorie malnutrition

## Abstract

Background

Malnutrition develops when there is an inadequacy of one or more than one macronutrient for optimum body functioning. This study was designed to determine the effectiveness of ready-to-use therapeutic food (RUTF) in children with protein-calorie malnutrition (PCM) in terms of weight gain and mid-upper arm circumference (MUAC) improvement.

Methodology

This prospective observational study was done at The Department of Pediatrics, National Institute of Child Health, Karachi, Pakistan from 1^st^ January 2021 to 31^st^ December 2021. A total of 159 children of either gender between six and 59 months of age and diagnosed with PCM were included. All children participating in the study were asked to come back for a follow-up every two weeks until they are 12 weeks old. Outcomes were measured in terms of comparison of weight gain and MUAC at baseline and after 12 weeks of RUTF.

Results

Of the 159 children, 94 (59.1%) were female. The mean age was 16.8±9.2 months, and 88 (55.3%) children were aged between six to 12 months. The mean body weight was 6.8±9.7 kg. The mean MUAC was calculated to be 116±9.1 mm. There were 121 (76.1%) children who had severe acute malnutrition. One hundred and thirty-one children completed the planned 12-week of follow-up using RUTF in this study, so they were included in the final analysis. Body weight increased significantly from baseline to 12-weeks after RUTF (6.8±1.7 kg vs. 7.6±1.2kg, p<0.0001). Likewise, MUAC also increased from baseline to 12 weeks after RUTF (116.0±9.1 mm vs. 127.2±8.2 mm, p<0.0001).

Conclusion

The RUTF was found to significantly improve weight gain and MUAC among children aged between six to 60 months with PCM during a period of 12-weeks. Early diagnosis and timely intervention can improve outcomes among children with PCM. Community-based interventions can be aimed to improve the nutritional status of children in a developing country like Pakistan.

## Introduction

Malnutrition develops when there is an inadequacy of one or more than one macronutrients in the body for optimum body functioning [1]. Children below five years of age, pregnant and lactating women, and older adults are most frequently affected by malnutrition. Among children below five years of age, around 165 million are estimated to be affected with malnutrition worldwide. Malnutrition is also estimated to influence around 50% of all pediatric age group deaths globally [2-4]. Malnutrition is a multifactorial entity that encompasses various clinical statuses including protein-calorie malnutrition (PCM) [5].

The PCM among children is a global health issue mainly in developing countries [6]. The United Nations International Children's Emergency Fund (UNICEF) recognized environmental, economic, and socio-political factors as the main causes behind malnutrition while poverty is considered to be the most important root cause behind malnutrition [7,8]. Pakistan has been reported to be among the nations with the highest proportion of childhood malnutrition especially PCM when compared to other developing counties [9,10].

The ready-to-use therapeutic foods (RUTF) were developed from the existing liquid F-100 diet recommended by WHO for treating children with malnutrition including PCM [11]. The RUTF does not need water for preparation, has a sweet palatable taste, and is shelf-stable for up to a duration of two years [12]. Different studies were carried out to determine the effect of RUTF on malnutrition and reported the recovery with respect to weight gain, such as the Pakistani study by Khan et al. that reported weight gain in 81.8% of malnourished children [11]. Another study by Sigh et al. reported weight gain in 40.5% of malnourished children treated with RUTF [13].

Many studies have been conducted throughout the world to measure the response of RUTF in children with PCM but local data is scarce regarding this, especially in the population of the Sindh Province. Therefore, this study was done to determine the effectiveness of RUTF in children with PCM in terms of weight gain and mid-upper arm circumference (MUAC) improvement.

## Materials and methods

This prospective observational study was done at the Department of Pediatrics, National Institute of Child Health, Karachi, Pakistan from 1st January 2021 to 31st December 2021. The sample size of 159 was calculated using the proportion of Khan et al. who reported the weight gain in 81.8% of malnourished children treated with RUTF [11], by taking a confidence interval of 95% and a margin of error of 6%. Inclusion criteria were children of either gender aged six to 59 months diagnosed with PCM. Exclusion criteria were children diagnosed with secondary malnutrition or suffering from uncontrolled systemic infections, severe cerebral palsy, or mental health problems.

Approval from Institutional Ethical Review Board was acquired (IERB-08/2021, dated: 04-11-2021) for this study. Informed and written consent was sought from parents/guardians of all study participants. Detailed medical history of each patient including name, gender, age (months), weight (Kg), height (cm), and MUAC (cm) were obtained. The RUTF was advised in all children depending upon children's body weight based on an estimated average requirement of 200 Kcal/Kg/day. All children participating in the study were asked to come back for a follow-up visit after every two weeks until they completed 12 consecutive weeks. All the data was recorded in proforma by the researcher.

The PCM was categorized as mild acute malnutrition (MAM), diagnosed either by MUAC between 11 to 12.5 cm or weight for height z score (WHZ) between -2 and -3 standard deviation (SD) or severe acute malnutrition (SAM), diagnosed either by MUAC < 11.5 cm or WHZ < - 3 SD. Mid-upper arm circumference (MUAC) was measured conventionally over the left upper arm of children by using MUAC tape. The WHZ was calculated by using WHO tables for boys and girls. The RUTF was vitamin and mineral fortified peanut paste food mixed with dry milk products for the treatment of malnutrition. Outcomes were measured as a comparison of weight gain and MUAC at baseline and after 12 weeks of RUTF.

For data analysis, Statistical Package for Social Sciences (SPSS) version 26.0 (IBM Corp., Armonk, NY, USA) was employed. Mean and SD was calculated for quantitative variables whereas frequency and percentages were calculated for categorical data. Effect modifiers like gender, age, and type of protein-energy malnutrition (PEM) were controlled by stratification with weight gain and MUAC improvement. Body weight and MUAC at baseline and after 12 weeks of RUTF were compared by applying paired sample t-test. A p-value below 0.05 was considered statistically significant.

## Results

Of a total of 159 children, 94 (59.1%) were female. The mean age was 16.8±9.2 months while 88 (55.3%) children were aged between six to 12 months. The mean body weight was 6.8±9.7 kg. The mean MUAC was calculated to be 116±9.1 mm. There were 121 (76.1%) children who had SAM. The area of residence was rural with 101 (63.5%) children. Maternal education status was illiterate in 45 (28.3%). Exclusive breastfeeding in the first six months was noted in 25 (15.7%) cases. Table 1 shows the baseline characteristics of all children enrolled in this study.

**Table 1:** Baseline characteristics of children (n=159)

Characteristics			Number (%) / Mean±SD
Gender		Male	65 (40.9%)
		Female	94 (59.1%)
Age in Months		6-12	88 (55.3%)
		13-24	42 (26.4%)
		25-60	29 (18.2%)
Age in Months			16.8±9.2
Weight in Kg			6.8±9.7
Mid-Upper Arm Circumference in mm			116±9.1
Types of Protein Calorie Malnutrition	Severe Acute Malnutrition		121 (76.1%)
	Mild Acute Malnutrition		38 (23.9%)
Area of Residence		Rural	101 (63.5%)
		Urban	58 (36.5%)
Monthly Family Income (Pakistan Rupees)		<18,000	83 (52.2%)
		18,000 to 40,000	65 (40.9%)
		>40,000	11 (6.9%)
Maternal Education		Illiterate	45 (28.3%)
		Literate	114 (71.7%)
Exclusive Breastfeeding in 1^st^ 6 months			25 (15.7%)
Appropriate Complimentary Breastfeeding			31 (19.5%)
Good Hygiene Practice			13 (8.2%)

Twenty-five children discontinued follow-ups while mortality was reported in three, so these 28 children were left out from the final analysis. One hundred and thirty-one children completed the planned 12-weeks of follow-up using RUTF in this study and so they were included in the final analysis. Body weight increased significantly from baseline to 12 weeks after RUTF (6.8±1.7 kg vs. 7.6±1.2kg, p<0.0001). Likewise, MUAC also increased from baseline to 12 weeks after RUTF (116.0±9.1 mm vs. 127.2±8.2 mm, p<0.0001). Table 2 shows the comparison of outcome measures among study participants from baseline to 12 weeks after RUTF.

**Table 2:** Outcome measures among study participants from baseline to 12 weeks after RUTF

Outcomes	At Baseline (n=159)	At 12 weeks after RUTF (n=131)	P-Value
Body Weight in kg	6.8±1.7	7.6±1.2	<0.0001
Mid-Upper Arm Circumference in mm	116.0±9.1	127.2±8.2	<0.0001

## Discussion

Malnourished children frequently look very thin or wasted with malnourishment being a common cause of morbidity and mortality among pediatric age groups [14,15]. In resource constraint healthcare settings, treatment of malnourishment is not always attainable due to practical reasons while a home-based therapeutic approach is commonly considered a better option for these children. The RUTF is made as per standard guidelines as described by WHO which satisfies the daily energy requirements of malnourished children [16].

In this study, we noted that a net increase of 0.8±0.5 kg body weight was recorded among study participants with PCM, and the difference in body weight at the end of the study period, when compared to the baseline body weight, was statistically significant (p<0.0001). Likewise, MUAC was also observed to have a net increase of 11.2±0.9 mm (p<0.0001). A recent study from Pakistan enrolling 91 children with SAM under the age of five years revealed that RUTF not only influenced significant weight gain among malnourished children but also improved the developmental potential of the malnourished children [17]. These findings are also consistent with what has been found in other studies conducted in Ethiopia, Pakistan, and Ghana [16,18,19].

Although data exists regarding various interventions done among children with malnutrition not much research has been performed to evaluate the efficacy of RUTF among PCM for 12 weeks in Southern Asia. Studies have shown that a direct relationship exists between children's development and nutritional status [20,21]. Malnourished children are observed to lack development in all main domains of pre-treatment screening while inappropriate nutritional supply can be a cause of various abnormalities like reduction in brain size, abnormal impact on cell maturation that can result in behavioral disorders with delayed personal and social development that can also occur due to inadequate nutritional supply [20,22].

In this study, we observed that 81.7% of children with PCM were aged less than two years. Exposure to malnourishment in the infancy period is thought to cause a high risk of developmental delay in the later years of life as infancy and toddlerhood are known to be periods of rapid development and are influenced by socio-demographical and environmental issues [17,22]. For the treatment of malnourished children, community-based interventions for addressing acute malnutrition like RUTF is effective. The RUTF has been found to fulfill micronutrient and macronutrient needs required for catch-up growth and appropriate developmental goals so timely recognition of PCM and its treatment can result in satisfactory weight gain and increase in MUAC as was observed in this study. On the other hand, delay in the recognition and treatment of PCM can progress into irreparable damage [23,24]. The WHO endorses timely utilization of RUTF for the attainment of required weight gain and satisfactory recovery among malnourished children [25]. The current study showed that the 12-week treatment with RUTF resulted in significant gains in mean weight and MUAC among children with PCM.

As this was a single-center study conducted with relatively small sample size, our findings cannot be generalized. High rates of loss of follow-up in the present study could have also influenced the outcomes as it is presumed that children with poor weight gain might have stopped follow-up and consulted some other childcare facilities. As the present study had a relatively small follow-up duration of 12 weeks, children having RUTF must be followed up for a longer duration so that the long-term effects of these interventions are determined. Ideally, we should have analyzed mico-nutrient levels in the body to further strengthen our beliefs about the role of RUTF among children with PCM but due to financial and administrative limitations, we could not do that.

## Conclusions

The RUTF was found to significantly improve weight gain and MUAC among children aged between six to 60 months with PCM during a period of 12 weeks. Early diagnosis and timely intervention can improve outcomes among children with PCM. Community-based interventions can be aimed to improve the nutritional status of children in a developing country such as Pakistan. There might even be potential for community-targeted interventions to improve the overall nutritional level of children.

## References

[1] Jackson AA (2018). Identifying children at risk of malnutrition. Nutr J.

[2] Grellety E, Golden MH (2018). Severely malnourished children with a low weight-for-height have a higher mortality than those with a low mid-upper-arm-circumference: I. Empirical data demonstrates Simpson's paradox. Nutr J.

[3] Ansuya Ansuya, Nayak BS, Unnikrishnan B, George A, N SY, Mundkur SC, Guddattu V (2018). Risk factors for malnutrition among preschool children in rural Karnataka: a case-control study. BMC Public Health.

[4] Zhang Y, Huang X, Yang Y (2018). Double burden of malnutrition among children under 5 in poor areas of China. PLoS One.

[5] Kumari P (2018). Prevalence of protein energy malnutrition among under-five children belonging to rural areas of Ambala, Haryana, India. Res Rev J Med.

[6] James WP (2018). From treating childhood malnutrition to public health nutrition. Ann Nutr Metab.

[7] James WP (2017). A clinical nutritionist's experience and expectations. Eur J Clin Nutr.

[8] (2022). 2018 global nutrition report reveals malnutrition is unacceptably high and affects every country in the world, but there is also an unprecedented opportunity to end it. https://www.unicef.org/press-releases/2018-global-nutrition-report-reveals-malnutrition-unacceptably-high-and-affects.

[9] Asim M, Nawaz Y (2018). Child malnutrition in Pakistan: evidence from literature. Children (Basel).

[10] Laghari ZA, Soomro AM, Tunio SA (2015). Malnutrition among children under five years in district Sanghar, Sindh, Pakistan. Gomal J Med Sci.

[11] Khan S, Iqbal MI, Ali I, Fawad U, Arshad R, Ishfaq K (2018). Weight gain in malnourished children on who recommended therapeutic feeding formula f-100. Pak Pediatr J.

[12] Bazzano AN, Potts KS, Bazzano LA, Mason JB (2017). The life course implications of ready-to-use therapeutic food for children in low-income countries. Int J Environ Res Public Health.

[13] Sigh S, Roos N, Chamnan C, Laillou A, Prak S, Wieringa FT (2018). Effectiveness of a locally produced, fish-based food product on weight gain among Cambodian children in the treatment of acute malnutrition: a randomized controlled trial. Nutrients.

[14] Javan R, Kooshki A, Afzalaghaee M, Aldaghi M, Yousefi M (2017). Effectiveness of supplementary blended flour based on chickpea and cereals for the treatment of infants with moderate acute malnutrition in Iran: a randomized clinical trial. Electron Physician.

[15] Nangalu R, Pooni PA, Bhargav S, Bains HS (2016). Impact of malnutrition on pediatric risk of mortality score and outcome in Pediatric Intensive Care Unit. Indian J Crit Care Med.

[16] Aguayo VM, Badgaiyan N, Qadir SS, Bugti AN, Alam MM, Nishtar N, Galvin M (2018). Community management of acute malnutrition (CMAM) programme in Pakistan effectively treats children with uncomplicated severe wasting. Matern Child Nutr.

[17] Saleem J, Zakar R, Bukhari GM, Naz M, Mushtaq F, Fischer F (2021). Effectiveness of ready-to-use therapeutic food in improving the developmental potential and weight of children aged under five with severe acute malnourishment in Pakistan: a pretest-posttest study. Int J Environ Res Public Health.

[18] Akparibo R, Harris J, Blank L, Campbell MJ, Holdsworth M (2017). Severe acute malnutrition in children aged under 5 years can be successfully managed in a non-emergency routine community healthcare setting in Ghana. Matern Child Nutr.

[19] Kabalo MY, Seifu CN (2017). Treatment outcomes of severe acute malnutrition in children treated within outpatient therapeutic program (OTP) at Wolaita zone, Southern Ethiopia: retrospective cross-sectional study. J Health Popul Nutr.

[20] Chattopadhyay N, Saumitra M (2016). Developmental outcome in children with malnutrition. J Nepal Paediatr. Soc.

[21] Lelijveld N, Seal A, Wells JC (2016). Chronic disease outcomes after severe acute malnutrition in Malawian children (ChroSAM): a cohort study. Lancet Glob Health.

[22] Yakoob MY, Lo CW (2017). Nutrition (Micronutrients) in child growth and development: a systematic review on current evidence, recommendations and opportunities for further research. J Dev Behav Pediatr.

[23] Collins S (2004). Community-based therapeutic care: a new paradigm for selective feeding in nutritional crises. http://lib.riskreductionafrica.org/bitstream/handle/123456789/1426/community-based%20therapeutic%20care.pdf?sequence=1.

[24] Collins S, Sadler K, Dent N (2006). Key issues in the success of community-based management of severe malnutrition. Food Nutr Bull.

[25] (2022). Essential nutrition actions: improving maternal, newborn, infant and young child health and nutrition. https://apps.who.int/iris/handle/10665/84409.

